# The Cost of Cochlear Implantation: A Review of Methodological Considerations

**DOI:** 10.1155/2011/210838

**Published:** 2011-10-17

**Authors:** Costa Nadège, Garnault Valérie, Ferlicoq Laura, Derumeaux-Burel Hélène, Bongard Vanina, Deguine Olivier, Fraysse Bernard, Molinier Laurent

**Affiliations:** ^1^Department of Medical Information, University Hospital, 31059 Toulouse Cedex 9, France; ^2^UMR 1027, INSERM-University Paul Sabatier Toulouse III, 31073 Toulouse, France; ^3^Department of Epidemiology, Health Economics and Public Health, University Hospital, 31073 Toulouse, France; ^4^Department of Otolaryngology, University Hospital, 31059 Cedex 9 Toulouse, France

## Abstract

*Objectives*. Cost studies can provide useful guidance, so long as they adhere to accepted methodology. Cochlear implants (CIs) are electronic devices introduced surgically into the inner ear. It is a relevant example to review cost study analyses because of its costliness. The aim of this study was to review relevant published cost studies of CI to analyze the method used. *Methods*. First, we described the key points of cost study methodology. Cost studies relating to CI were systematically reviewed, focussing on an analysis of the different methods used. *Results*. The methods, data sources, and estimated cost categories in each study varied widely. The paper showed that cost studies adopted significantly different approaches to estimate costs of CI, reflecting a lack of consensus on the methodology of cost studies. *Conclusion*. To increase its credibility, closer agreement among researchers on the methodological principles of cost studies would be desirable.

## 1. Introduction

Cochlear implants are electronic devices introduced surgically into the inner ear. These implants restore useful hearing to profoundly or totally hearing-impaired patients. There is no comparable alternative medical treatment for profound-total deafness. Unlike hearing aids, cochlear implantation (CI) necessitates a surgical procedure and incurs substantial costs throughout the lifetime of the recipient. In actual practice, the rehabilitation process has to be continued for several years, especially in children [1].

CI has taken an important rise in many countries in the last twenty years. Decision makers in charge of public health are faced with the decision of whether to include cochlear implants in the basic medical benefit package. In the face of scarce resources, decision makers are not only interested in the effectiveness of certain healthcare interventions but also in the costs that are involved. Several studies have analysed the costs of cochlear implants, in particular in the United Kingdom [2–14], United States [15–22], Australia [23], France [24], The Netherlands [25], Germany [26], Belgium [27], and Asia [28, 29], and some of these studies have shown the costs of CI for healthcare systems, leading to major rethinking in the field of health cost rationalization. Healthcare financing conditions are fitted to these countries, and differences in healthcare settings influence the results of a cost analysis.

Cost study aims to describe the economic burden of a specific disease to society. They are designed to evaluate not only the costs attributable to the treatment of a particular illness but also to evaluate actual illness-related global costs [27]. In principle, they should either inform the most accurate choices in resource allocation or be used in full economic evaluations of healthcare programmes and treatments [30, 31]. Cost studies have been criticised for not really providing useful information or enabling choice of priorities [32, 33]. It can however play an important role in informing cost estimates for use in further economic evaluations [34, 35]. These studies should be carried out in accordance with a clear and widely accepted methodology [34, 36].

The aim of this study was to review relevant published cost studies of cochlear implant to analyze the method used. First, we provided a general description of the cost study method. We then systematically reviewed the studies on costs relating to cochlear implantation, analyzing the different methods used.

## 2. Methods

### 2.1. Cost Study

To conduct a cost study, it is necessary to first define the pathological state, the epidemiological approach, the type of costs to be assessed, and, thus, the perspective of the study. Subsequently, data on resource consumption and unit costs can be gathered, and results presented and methodically discussed, in conjunction with sensitivity analysis to test their robustness.

#### 2.1.1. Defining the Disease and the Patient

The costs attributable to an illness depend widely on how the disease is defined diagnostically. Studies habitually use the International Classification of Disease (ICD 10th). Cost study should precisely define the disease stage investigated, including the identification of subgroups of patients according to clinical and economical criteria. This makes the analysis more precise and relevant.

#### 2.1.2. Perspective of the Analysis and Costs Assessed

Different types of costs (direct, indirect, and intangible) are included in economic evaluations, depending on the study's point of view. For example, when the healthcare system perspective is taken, only direct healthcare costs incurred by the payer (National Health Insurance) are considered. Instead, when a societal point of view is taken, indirect costs and the “out-of-pocket” for patient and family must also be included.

#### 2.1.3. Estimating Resource Consumption

Methods for estimating resource consumption vary depending on the availability of the data.

A cost study can be prospectively or retrospectively performed depending on the temporal relationship between the initiation of the study and the data collection.

In prospective cost studies, the relevant events have not already occurred when the study is initiated. The process of data collection needs to be done by followingup a sample of patients over the study period. This approach usually uses medical records, data collected during clinical trials, and questionnaires to patients.

Conversely, in retrospective cost study, all the relevant events have already occurred when the study is initiated. The process of data collection must refer to data already recorded.

For this approach, the activity data can be collected either using aggregate figures from hospital admissions, consultations, mortality, and so forth, (“top-down” method), or by referring to the record of a sample of patients (“bottom-up” method).

A prospective approach (e.g., from medical records or clinical trials) is preferred to a retrospective approach because of the bias risk and the quality data [37].

#### 2.1.4. Valuation of Unit Costs

Costs should represent the value of the input in its best alternative use, that is, the opportunity cost. In a well-functioning, competitive market, this would be the minimum price required to use the input in its current use rather than in an alternative use [31, 38].

Direct costs measure the resources used to treat an illness. It can be estimated in many ways depending on the study design. These methods include per capita expenditures, national tariffs, market prices, data from published studies, and specific estimates. Patient's charges and tariffs do not generally give an accurate estimate of the underlying costs. Market price can be used to value some costs categories like drugs and rehabilitation items (e.g., eyeglasses, hearing aids, etc.). Data published in studies conducted in other countries are biased to the healthcare system considered, and to local medical practice in the country where the study was conducted. Therefore, the results are unlikely to be transferable from one setting to another. The use of costs estimated by some healthcare centres or hospitals, based on an accounting principle, is another way to value direct costs. However, since many costs may be hard to attribute directly to specific items (e.g., overhead), methodology should be clearly specified.

Medical care costs are characterized by certain distributional properties (e.g., analytical accounting). Failure to account for these issues will result in a biased estimate of costs and misguided conclusions of the study. Censoring data is recognised as an issue that can bias cost studies.

Indirect costs measure lost productivity, that is, the effect of the illness on the ability of either patients or their caregivers to work (e.g., lost income) or engage in other activity (e.g., cleaning the house). Two methods are usually used to value indirect costs. The human capital approach (HCA) is mainly used and based on the principle of productive potential [39, 40]. This method measures the lost production, in terms of lost earnings, of a patient or caregiver and often includes the value of household work, usually valued as the opportunity cost of hiring a replacement from the labour market. Friction cost method (FCM) assumes that, in the absence of full employment, indirect costs occur only during the time necessary to restore the initial level of production (e.g., friction period) by replacing the sick worker or by reorganizing the production process [30, 31]. When production losses because of mortality are not considered, future lost earnings are neglected [40, 41]. 

Productivity costs estimated by the HCA were more than three times higher than the productivity costs estimated by the FMC [42]. HCA overestimates the magnitude of indirect costs. But FMC is seldom applied as it requires a huge amount of information.

Educational costs in implanted children measure the placement costs in an educational setting and the support costs provided by language pathologists, educational audiologists, special educators, teachers of the hearing impaired, interpreters, occupational therapists, and instructional assistants [17]. Cost of placement in an educational setting is often assessed through public education budge, and support costs are valued through salary costs of public and special school [3, 17, 19, 26].

#### 2.1.5. Discounting Costs

Discounting is an economic method that captures an individual's preference for income today rather than income in the future and is frequently applied when cost studies are considered over several years. In the UK, the choice of a discount rate originates from the social opportunity cost approach, but it has increasingly been viewed as an interest psychological rate of the society [31].

In the USA, the public health service panel on cost effectiveness in health and medicine estimates that the most appropriate discount rate is 3% [43].

The following equation is applied to estimate costs:

(1) $$C_{a}=C_{t}\overset{t}{\underset{n=1}{\sum }}{\left(1+r\right)}^{-n},$$

where *C*_*a*_ present value of cost strategy, *C*_*t*_ is value of cost strategy in year *t*, *r* is discount rate, and *t* is time period.

#### 2.1.6. Sensitivity Analysis

A sensitivity analysis is recommended in cost studies that contain a certain degree of uncertainty. This type of analysis tests the robustness of the results by varying in a range of key variables (e.g., prevalence or incidence rate, discounting rate, unit costs, survival probabilities, etc.). A sensitivity analysis can take various forms: simple, multiway, and probabilistic [44]. For cost studies, it seems particularly interesting to do sensitivity analysis using different methods for estimating types of cost. Also, it seems more credible for health policy analysis to present the cost studies as a range of possible costs.

#### 2.1.7. Presentation of Results

The presentation of cost study results should be consistent with collected data and should break down results into as many components as possible with full explanation given for clarity.

### 2.2. Literature Review

#### 2.2.1. Study Selection

**Figure 1:**
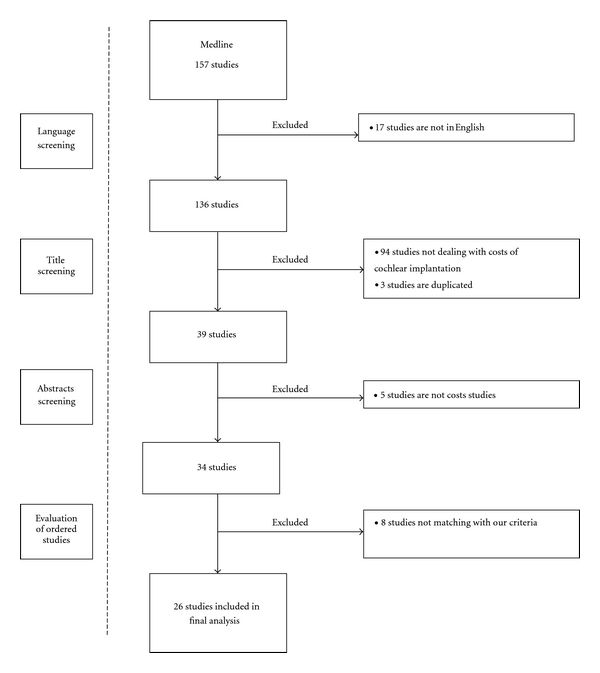
Literature search and selection process.

A bibliographic search was performed on an international medical literature database (Medline, from 1966 until April 2011). All studies published in English that assessed costs of CI were selected. Five combinations using keywords were carried out (“cochlear implant” AND “cost study” OR “cochlear implant” AND “cost analysis” OR “cochlear implant” AND “cost evaluation” OR “cochlear implant” AND “economic evaluation” OR “cochlear implant” AND “economic analysis”). The results of this search provided us with 157 studies, 136 of which were in English language. On these 136, 94 studies did not deal with costs of cochlear implantation and 3 studies were duplicated. Forty two abstracts were firstly selected, 37 of them underwent a subsequent full paper reading, thus providing 26 papers.  Figure 1 illustrates the literature search and selection process and presents reasons for excluded studies.

Our aim was to assess the methods adopted by the authors rather than to compare cost estimates.

#### 2.2.2. Study Review

A systematic review was performed. One author (N. Costa) selected abstracts. Five methodologists (N. Costa, H. Derumeaux-Burel, L. Ferlicoq, V. Garnault, and L. Molinier) each read the 37 papers retrieved by the search strategy and reviewed the 26 selected papers. L. Molinier did not participated in the analysis to the study he previously published “The economics of cochlear implant management in France: a multicentre analysis” [24]. In keeping with the key methodological points identified in the first part of the paper, they asked questions based on existing checklists for full economic evaluations [32, 45]. An equal weight was given to each item. The final score was the sum of the 13 individual items. The objective was not to establish a hierarchy in the criteria used by allocating them different weights, but to use these criteria to analyse the methods used. Each study was assessed separately by the reviewers. Finally, a meeting to review the outcome was called, and a consensus was reached by discussion. For each item, an agreement between the reviewers was found. Then, all authors, both clinicians and methodologists, discussed the results.

## 3. Results

Twenty six studies met our criteria (Table 1). Sixteen studies were carried out in Europe [2–7, 9, 10, 12–14, 24–27], 7 in North America [7, 15–17, 19, 20, 22], 2 in Asia [28, 29], and 1 in Australia [23].

Eight studies were cost analysis studies [2–4, 9, 19, 24, 25, 27], and 18 were global economic evaluations, including 16 cost-utility analyses [5–8, 10, 12–16, 20–23, 28, 29] and 2 costs-benefit analysis [17, 26].

Nineteen studies selected a sample ranging in size from 8 to 403 patients [2–5, 9, 12–17, 19–25, 27, 28]. Three studies modelled costs without including patients [6, 7, 13].

### 3.1. Defining the Disease and Population

Cochlear implants are devices that are indicated to treat severe to profound deafness. Implantation can be done unilaterally (i.e., one ear) or bilaterally (i.e., both ears). The indications of CI depend widely on deafness severity and children or adults recipients.

Seven studies were performed on adults [12, 14, 20–22, 27, 28]. Among these, 5 defined the deafness as profound [12, 14, 21, 22, 27], one as severe to profound [20], and one did not specify the severity of the deafness. Three studies indicated the nature of implantation, bilateral for Vantrappen et al. [27], unilateral for the UK CISG [14], and both for Summerfield et al. [12]. No studies have used the ICD 10th for defining the disease. This classification lacks of precision for defining hearing loss and the indications of CI.

Thirteen studies were performed on children [2–5, 7–9, 13, 16, 17, 19, 25, 26]. Five defined the deafness as profound [8, 9, 16, 17, 19], one as severe [26], one as moderate [4], and six studies did not specify the level of deafness [2, 3, 5, 7, 13, 25]. Two studies indicated the nature of implantation, unilateral for Barton et al. [2] and unilateral and bilateral for Summerfield et al. [13].

Six studies included an assessment of cochlear implantation costs in both adults and children [6, 10, 15, 23, 24, 29]. Three defined the deafness as severe to profound [6, 15, 24], one as partial or profound [23], one as profound [10], and one did not specify the level of deafness [29]. Bond et al. indicated the unilaterally and bilaterally nature of implantation [6].

### 3.2. Perspective of the Analysis and Costs Assessed

Six studies did not specify the viewpoint adopted [3, 7, 17, 19, 21, 22]. Ten studies, including 8 European studies, adopted the healthcare payer's perspective [2, 6, 8, 12–14, 20, 23, 24, 26]. The service provider perspective was used in 5 studies [10, 15, 27–29] and the family perspective in 2 studies [4, 9]. The costs analysis was performed from the societal point of view in 3 studies [5, 16, 25].

Thirteen studies quantified only the direct medical costs [2, 9, 10, 12, 13, 15, 20–23, 26, 27, 29]. Direct costs considered by most studies included preoperative assessment, surgery, implant device, and followup (maintenance and rehabilitation). 

Two studies quantified both direct medical and nonmedical costs, limited to travel costs [24, 25]. Molinier et al. [24] estimated that travel costs accounted for 7% of total direct costs over one year. Severens et al. [25] have not broken up direct nonmedical costs per year.

Six studies [6–8, 17, 19, 26] estimated direct costs and educational costs. Four studies assessed education costs with a time horizon of one year [7, 8, 16, 19], while Schulze-Gattermann et al. assessed these costs during the school lifetime.

Four studies evaluated both direct and indirect costs [4, 5, 9, 16]. Cheng et al. [16] estimated travel costs and education costs as indirect costs. All of these estimated indirect costs in terms of loss productivity.

Barton et al. [3] only estimated the education costs.

### 3.3. Estimating Resource Consumption

Thirteen studies estimated resource consumption retrospectively [2–5, 9, 12, 14–17, 26–28]. 

Nine studies used a bottom up approach to gather activity data [2–4, 9, 15–17, 26, 27]. In the German study [26], data on resource utilisation were collected from patient questionnaire and four existing databases. The clinical records of 16 patients were analyzed in the study of Bichey et al. [15]. Two studies analyzed all procedures of each patient in one and twelve hospitals, respectively, through hospital database [2, 27]. Barton et al. [2] used also the clinical case notes of each patient. All claims submitted to Medicare were analyzed for 78 patients in the study of Cheng et al. [16]. Barton et al. have recorded educational data from teacher's questionnaires of 368 children [3], and they recorded the amount of out-of-pocket, lost productivity, and transfer payment (government benefit) from parent's questionnaires of 468 children [4]. To estimate educational data of 27 children, Francis et al. [17] used parents' interviews, school consultation, and individualized education plan. Parents of 216 children were interviewed with semistructured face-to-face interview in one American study [9], in order to record travel time and mode, lost productivity, and leisure time. 

Four studies used a top-down approach [5, 12, 14, 28]. Resource consumption was estimated using mainly published national indicators, data from national survey, published study, and by expert advice. The use of top-down approaches to assess resources consumption implies aggregate data processing, which, if not properly performed, could induce errors and unrealistic results. It was not always obvious to determine how data on resource consumption were processed to obtain more detailed data. To estimate resource utilisation, Barton et al. [5] used published sources, and Lee et al. [28] used hospital data and provided estimates based on the references by two otology physicians. Two studies [12, 14] have chosen to create a common profile of patient care and to combine it with the date of implantation of each patient.

Four studies estimated resource consumption prospectively [10, 20, 24, 25]. Molinier et al. [24] recorded data through patient questionnaire for 306 children and 254 adults and Palmer et al. [20] through a diary for 40 adults. In two studies [10, 25], time spent on various activities related to implantation was collected by the several professionals involved in cochlear implantation.

Six studies [6, 7, 13, 21–23] used decision model and estimated resource consumption mainly through published sources.

Two studies did not precise the approach used to gather activity data [8, 19]. One study [8] used published national sources, and another one [19] used questionnaire and published national sources to estimate resource consumption.

The studies collected information over various periods. The follow-up period was the lifetime period (i.e., time to implantation until death) in 13 studies. Five of these studies included only children [2, 5, 7, 8, 16], five included only adults [12, 14, 20–22], and 3 included both children and adults [6, 13, 29]. Lifetime period ranged from 71 to 73 years for children and ranged from 21 to 33 years for adults. The one-year follow-up period was considered in 5 studies. Three of these studies included only children [4, 9, 19], and one included adults [3, 17, 26]. This period ranged from 12 to 15 years. Three studies chose a followup of 5 years for children [25], 12 years, and 20 years, respectively, for both adults and children [10, 23]. Three studies did not specify the follow-up period [15, 28].

### 3.4. Valuation of Unit Costs

Different methods, mainly extrapolations from national sources and published data, were adopted to assess direct costs. One American study estimated costs mainly from Medicare payments and from wholesale prices for implant device [16]. A German study [26] valued direct medical costs from answers resulting from the parents' questionnaire and from the Medical University of Hanover accounting database. A French study [24] assessed hospital costs with the French DRGs and the national unit cost scale, implant device with retail prices, and other costs with the appropriate reimbursement tariffs used by French Social Health Insurance. No information relating to the valuation of unit costs was reported in the Chinese study [29]. Three studies [12, 15, 22] took into account costs calculated in hospitals. Summerfiel et al. [12] used also retail prices for implant device. Lee et al. [28] used normative costs, which two otology physicians estimated based on references and hospital data. Palmer et al. [20] recorded actual charges on one hospital and physician bills that were obtained from study participants. When bills were not available, they used the price based on the 1996 Medicare maximum allowable charge and the Red Book wholesale drug price. Francis et al. [17] estimated direct costs based on 1997 cost data from The Listening Center. The method of microcosting, in which a unit cost is derived for each resource, was used in two studies [2, 27]. Vantrappen et al. [27] used also the reimbursement of CI device by social health insurance. In The Netherlands study [25], cost was estimated through price of hospital day in one center, retail prices for implant device, and reimbursement prices for diagnostic tests. Price of hospital day was obtained by dividing the total annual cost by the actual number of hospitalization days in the Ear, Nose and Throat Department. An English study [14] determined costs with the UK NHS data. Two studies [4, 9] assessed direct non medical costs incurred by families as out-of-pocket expenditure and travel costs by using the value estimated by respondents.

Several studies have quantified educational costs. Cost data for two American studies [17, 19] were derived from the State of Maryland Department of Education Budget and from the salary costs of public schools without overhead. The German study [26] used public authorities' charges to estimate educational costs. Barton et al. [3] assessed educational costs through a UK government report (Department for Education and Skills, 2002) for nursery, primary, and secondary schools, and a UK School inspection reports for special schools and schools for deaf children. They valued support costs through the budgets that special education UK services devote to children with impaired hearing.

Four studies quantified indirect costs [4, 5, 9, 16]. Three studies have valued lost of parents productivity with the HCA. Cheng et al. [16] used the parents' wage until their children would be aged 18 years. Two other studies [4, 9] used an average gross weekly wage rate, derived from the New Earning Survey 2002, minus 35% to take account of tax, pension, and national insurance. These two studies took into account leisure time, but they did not value this time. One study quantified change in future earning for children [16], by taking into account differences in school placement and nondeaf and deaf employment rates and wages. Sach et al. valued children's missed schooling using published estimate of lifetime earnings per hour of special education [9].

### 3.5. Discounting Costs

Costs were discounted in 22 studies. The discount rate chosen was 6% for 7 studies [2, 7, 8, 10, 12, 14, 26], 5% for six studies [17, 20–23, 25], 3, 5% for two studies [6, 13], and 3% for four studies [3–5, 28]. One study discounted costs with two discount rates, 3 and 5% [16]. One study did not specify the rate value.

In two studies where costs were not discounted, the time horizon was short (=1 year in both studies) [9, 24].

### 3.6. Sensitivity Analysis

Fifteen studies [3, 4, 6, 8, 9, 13, 14, 16, 20–23, 26, 28] performed a sensitivity analysis and discussed the variables that had a significant impact on cost estimates. Many key variables were identified. Two variables were particularly analyzed in these studies. Nine studies [2, 6, 8, 12, 14, 21–23, 28] analyzed the discount rate variation, and six [6, 8, 14, 20–22] analyzed the variation of the period during which cochlear implant was used. Two studies [7, 17] performed a sensitivity analysis without defining the key variables.

### 3.7. Presentation of Results

Most studies presented results clearly. They were mainly well explained and consistently set out in relation to the methods adopted. Nevertheless, one study [29] presented results without defining the cost analysis method. Eight studies [5, 15–17, 19, 26, 28, 29] did not sufficiently disaggregate costs, thus reducing the strength of the information provided. 

All studies presented results in terms of cost per patient. One English study [3] proposed also the total health-service costs of implanting new children and providing followup to children already implanted.

According to the key methodological points, we have drafted a checklist of questions related to the seven items analyzed (Table 2). For 12 studies [2–4, 6, 12–14, 16, 23–26, 35], the answer of seven to ten questions was “yes.”

This checklist was developed on the model described by Drummond et al. [31] and adapted to the cost studies by Molinier et al. [46].

## 4. Discussion

This study reviews 26 cost studies on cochlear implantation with the main goal of analyzing the various methodologies. According to the key methodological points, three studies were scored “yes” on the majority of the questions [2, 3, 12, 13, 16, 23–26]. Three studies scored “yes” to any questions [4, 6, 14].

Studies analyzed here confirm that cochlear implantation is costly the first year after implantation. 

Nevertheless, commenting on these quantitative results is problematic because significantly different approaches have been adopted to estimate the costs of cochlear implantation. There is also marked differences in the types of costs included and the sources used to assess activity data. Therefore, the comparison of the results reported in each study is not very useful. The methods used to estimate CI costs vary widely across studies in the literature, which is probably due to the lack of consensus on the methodology. Therefore, the definition of standards, with a large consensus in the methodology selected to conduct these studies, should be a major concern for the scientific community [47, 48]. Bloom et al. propose in a first step to implement guidelines to standardized methods and study design for cost studies [49]. Nevertheless, we must bear in mind that, unlike clinical trial results, it is very difficult to generalize quantitative results of economic studies conducted in different countries. Economic results are difficult to compare on account of monetary issues, such as fluctuating exchange rates and different purchasing powers of currencies. Domestic characteristics also dramatically affect resource consumption and unit costs, including differences in clinical practice and healthcare system framework.

As several studies did not fully explain their methods, they were difficult to assess. This might be due to a general lack of economic awareness in the medical journals that support economic studies. Most of the studies reviewed were published in journals that did not demand sufficiently detailed and explicit explanations about the methodologies chosen. In 1996, the British Medical Journal published guidance information to authors and peer reviewers on economic evaluation, but it did not address costs studies [50]. A detailed description of the methodological choices would improve the credibility of cost studies.

## 5. Conclusion

Cost studies can provide information to support the political process as well as the management functions at different levels of healthcare organizations. These studies must be able to identify the actual clinical management of illness and to measure true costs.

Cost study results can serve as a baseline for further economic evaluations. Nevertheless, an insufficient description of methods may lead to misunderstandings. The cost studies of CI identified in this paper highlight the poor consensus of methodological approaches, perhaps reflecting a lack of stringency on the part of medical journals. Hence, journal should encourage researchers to give clear descriptions and discuss limitations, and a further effort should be made to validate methodology.

The viewpoint of the analysis must be stated. Resource consumption could be better estimated by the followup of a sample of patients, and unit costs of the facilities provided for patients' care could be carefully assessed.

## Figures and Tables

**Table 1:** Cost studies of cochlear implantation.

Study	Country	Type of healthcare system	Year of valuation	Study	Perspective	Design of cost analysis	Patients	Type of implantation	Discounted rate	Followup (year)	Mean direct medical costs (€)	Assessment	Implantation	Implant device	Followup	Educational costs (€)	Indirect costs
Barton et al. [2]	United Kingdom	NHS	1998-1999	Cost study	Health care payer	Retrospective multicentre	Children	Unilateral	6%	73	49, 859	3.743	27.863	23.281	18.253	No	No
Barton et al. [3]	United Kingdom	NHS	2001-2002	Cost study	Not specified	Retrospective	Children	Not	3%	12	No	No	No	No	No	Yes	No
Barton et al. [4]	United Kingdom	NHS	2001-2002	Cost study	Families	Retrospective	Children		3%	1	No	No	No	No	No	No	Yes
Barton et al. [5]	United Kingdom	NHS	2001-2002	Cost utility	Societal	Retrospective	Children		3%	From implantation to death	NA	NA	NA	NA	NA	Yes	Yes
Bichey et al. [15]	United States	Private insurance	NA	Cost utility	Service providers	Retrospective	Children/Adults		5%	NA	NA	821	23.284	15.408	NA	No	No
Bond et al. [6]	United Kingdom	NHS	2006	Cost effectiveness	Health care payer	Decision model	Children	Unilateral	3.50%	From implantation to death	39.075	3.189	20.311	16.405	10.257	Yes	No
Bond et al. [6]	United Kingdom	NHS	2006	Cost effectiveness	Health care payer	Decision model	Children	Bilateral	4%	From implantation to death	52.072	3.189	38.626	32.754	10.257	Yes	No
Bond et al. [6]	United Kingdom	NHS	2006	Cost effectiveness	Health care payer	Decision model	Adults	Unilateral	3.50%	From implantation to death	31.169	4.496	19.564	16.405	5.604	No	No
Bond et al. [6]	United Kingdom	NHS	2006	Cost effectiveness	Health care payer	Decision model	Adults	Bilateral	4%	From implantation to death	47.586	4.496	37.486	32.754	5.604	No	No
Carter and Hailey [23]	Australia	Public health insurance	1994	Cost utility	Health care payer	Decision model	Children		5%	20	29.528	639	14.488	12.607	9.860	No	No
Carter and Hailey [23]	Australia	Public health insurance	1994	Cost utility	Health care payer	Decision model	Adults		5%	20	22.228	639	14.488	12.607	4.863	No	No
Cheng et al. [16]	United States	Private insurance	1998–2000	Cost utility	Societal	Retrospective single center	Children		3% et 5%	73	NA	1.959	16.751	13.109	Not specified	Yes	Yes
Francis et al. [17]	United States	Private insurance	1998–1999	Cost study	NA	Retrospective	Children		5%	12 and 15	NA	NA	NA	NA	NA	Yes	No
Hutton et al. [7]	United Kingdom	NHS	NA	Cost effectiveness	Not specified	Decision model	Children		6%	70	18.788	1.139	21.684	Not specified	1.010	Yes	No
Koch et al. [19]	United States	Private insurance	1995-1996	Cost study	Not specified	NA	Children		Not defined	1	NA	NA	NA	NA	NA	Yes	No
Lee et al. [28]	South Korea	Private insurance	NA	Cost utility	Service providers	Retrospective single centre	Adults		3%	NA	NA	445	13.829	12.075	NA	No	No
Molinier et al. [24]	France	Public social insurance	2006	Cost study	Health care payer	Prospective multicentre	Children		NA	1	32.055	814	24.498	22.338	6.743	No	No
Molinier et al. [24]	France	Public social insurance	2006	Cost study	Health care payer	Prospective multicentre	Adults		NA	1	29.699	653	25.011	21.987	4.035	No	No
O'Neill et al. [8]	United Kingdom	NHS	1997-1998	Cost utility	Health care payer	NA	Children		6%	71	30.83	NA	NA	NA	NA	Yes	No
Palmer et al. [20]	United States	Private insurance	1994–1996	Cost utility	Health care payer	Prospective multicentre	Adults		5%	22	25.961	831	23.978	15.589	788	No	No
Sach et al. [9]	United Kingdom	NHS	2002	Cost study	Family	Retrospective single centre	Children		NA	1	No	No	No	No	No	No	Yes
Schulze- Gattermann et al. [26]	Germany	Public health insurance	1999	Cost benefit	Health care payer	Retrospective	Children		6%	From implantation to end of the school	NA	NA	NA	NA	NA	Yes	No
Severens et al. [25]	The Netherlands	Public and private insurance	1993–1996	Cost study	Societal	Prospective single centre	Children		5%	5	32.358	2.211	20.826	17.5	9.319	No	No
Summerfield et al. [10]	United Kingdom	NHS	1993–1996	Cost utility	Service providers	Prospective multicentre	Children		6%	12	27.228	1.678	19.226	14.730	6.348	No	No
Summerfield et al. [10]	United Kingdom	NHS	1992-1993	Cost utility	Service providers	Prospective multicentre	Adults		6%	12	24.331	1.126	19.45	14.73	3.773	No	No
Summerfield et al. [12]	United Kingdom	NHS	1997–2000	Cost utility	Health care payer	Retrospective multicentre	Adults	Unilateral	6%	30	26.954	3.236	19.845	15.691	3.872	No	No
Summerfield et al. [12]	United Kingdom	NHS	1997–2000	Cost utility	Health care payer	Retrospective multicentre	Adults	Bilateral simultaneous	6%	30	43.865	3.236	36.369	31.383	4.26	No	No
Summerfield et al. [12]	United Kingdom	NHS	1997–2000	Cost utility	Health care payer	Retrospective multicentre	Adults	Bilateral additional	6%	30	47.023	3.620	39.116	31.383	4.287	No	No
Summerfield et al. [13]	United Kingdom	NHS	2007	Cost utility	Health care payer	Decison model	Children	Unilateral	4%	From implantation to death	33.839	3.192	20.374	16.465	10.272	No	No
Summerfield et al. [13]	United Kingdom	NHS	2007	Cost utility	Health care payer	Decision model	Children	Bilateral	3.50%	From implantation to death	52.260	3.192	38.794	32.931	10.272	No	No
UK CISG et al. [14]	United Kingdom	NHS	1999	Cost utility	Health care payer	Retrospective multicentre	Adults	Unilateral	6%	21	37.632	406	32.346	28.637	4.88	No	No
Vantrappen et al. [27]	Belgium	Public social insurance	1994–1996	Cost study	Service providers	Retrospective single centre	Adults		NA	1	29.418	889	25.284	15.976	3.245	No	No
Wong et al. [29]	China	Public social	NA	Cost utility	Service providers	NA	Children		NA	66	NA	NA	NA	NA	NA	No	No
Wong et al. [29]	China	Public social	NA	Cost utility	Service providers	NA	Adults		NA	34	NA	NA	NA	NA	NA	No	No
Wyatt et al. [21]	United States	Private insurance	1990–1993	Cost utility	Not specified	Decision model	Adults		5%	33	24.023	1.251	21.938	13.772	841	No	No
Wyatt et al. [22]	United States	Private insurance	1993-1994	Cost utility	Not specified	Decision model	Adults		5%	23	24.972	1.328	22.810	14.031	841	No	No

All costs are in € (US$1 0,69 €, *£*1 1,126 €; May 31, 2011).

NA: Not Availaible, NHS: National Health Service.

No: No assessed.

**Table 2:** Answers to the methodological questions by study.

Question/answers		Was a clear definition of the deafness given?	Were the methods adopted carefully described?	Were activity data sources carefully described?	Were activity data appropriately assessed?	Were the sources of all cost values analytically described?	Were unit costs appropriately valued?	Were costs sufficiently disaggregated?	Were the major assumptions tested in a sensitivity analysis?	Were costs discounted?	Was the presentation of study results consistent with the methology of the study?	Total score by study
	Y	10	17	19	10	19	14	10	16	21	16	154
All studies	P	12	7	3	7	1	6	10	2	1	7	56
	N	4	2	4	9	6	6	6	8	4	3	53

	Y	Yes								Yes		4
O'Neill et al. [8]	P			P				P			P	3
	N		No		No	No	No		No			5

	Y	Yes	Yes	Yes		Yes		Yes	Yes	Yes	Yes	8
Summerfield et al. [12]	P				P		P					2
	N											0

	Y			Yes	Yes	Yes	Yes		Yes	Yes	Yes	7
Barton et al. [2]	P	P	P					P				3
	N											0

	Y	Yes	Yes	Yes		Yes	Yes	Yes	Yes	Yes	Yes	9
Severens et al. [25]	P											0
	N				No							1

	Y			Yes		Yes			Yes	Yes	Yes	5
Palmer et al. [20]	P	P	P		P		P	P				5
	N											0

	Y		Yes							Yes		2
Hutton et al. [7]	P	P							P		P	3
	N			No	No	No	No	No				5

	Y	Yes	Yes	Yes		Yes	Yes		Yes	Yes	Yes	8
Schulze-Gattermann et al. [26]	P				P			P				2
	N											0

	Y	Yes	Yes			Yes		Yes	Yes	Yes	Yes	7
Cheng et al. [16]	P			P	P		P					3
	N											0

	Y		Yes	Yes					Yes	Yes	Yes	5
Wyatt et al. [21]	P	P			P			P				3
	N					No	No					2

	Y								Yes	Yes		2
Lee et al. [28]	P		P					P			P	3
	N	No		No	No	No	No					5

Summerfield et al. [13]	Y		Yes	Yes		Yes	Yes		Yes	Yes	Yes	7
	P	P			P			P				3
	N											0

	Y	Yes		Yes	Yes	Yes	Yes	Yes				6
Vantrappen et al. [27]	P		P								P	2
	N								No	No		2

	Y									Yes		1
Wong et al. [29]	P											0
	N	No	No	No	No	No	No	No	No		No	9

	Y		Yes			Yes						2
Bichey et al. [15]	P	P					P	P				3
	N			No	No				No	No	No	5

	Y		Yes	Yes		Yes			Yes	Yes	Yes	6
Wyatt et al. [22]	P	P					P	P				3
	N				No							1

	Y	Yes		Yes	Yes	Yes	Yes			Yes		6
Summerfield et al. [10]	P		P								P	2
	N							No	No			2

	Y		Yes	Yes	Yes	Yes	Yes			Yes		6
Francis et al. [17]	P	P							P		P	3
	N							No				1

	Y		Yes	Yes	Yes		Yes	Yes	Yes	Yes	Yes	8
Carter and Hailey [23]	P	P				P						2
	N											0

	Y					Yes						1
Koch et al. [19]	P		P	P			P			P		0
	N	No			No			No	No		No	5

	Y	Yes	Yes	Yes	Yes	Yes	Yes	Yes	Yes	Yes	Yes	10
Barton et al. [4]	P											0
	N											0

	Y		Yes	Yes	Yes	Yes	Yes	Yes			Yes	7
Molinier et al. [24]	P	P										1
	N								No	No		2

	Y			Yes		Yes	Yes		Yes		Yes	5
Sach et al. [9]	P		P					P				2
	N	No			No					No		3

	Y		Yes	Yes						Yes	Yes	4
Barton et al. [5]	P	P			P							2
	N					No	No	No	No			4
	Y		Yes	Yes	Yes	Yes	Yes	Yes	Yes	Yes		8

Barton et al. [3]	P	P									P	0
	N											0

	Y	Yes	Yes	Yes	Yes	Yes	Yes	Yes	Yes	Yes	Yes	10
UK CISG [14]	P											0
	N											0

	Y	Yes	Yes	Yes	Yes	Yes	Yes	Yes	Yes	Yes	Yes	10
Bond et al. [6]	P											0
	N											0

Total score by study is the sum of answers.

P: partially; Y: Yes; N: No.
